# Vertical Migration of Adult Plecoptera and Trichoptera above Forested Headwater Streams

**DOI:** 10.3390/insects12090770

**Published:** 2021-08-27

**Authors:** Ruric O. Bowman, Robert F. Smith

**Affiliations:** 1 Department of Biology, Lycoming College, Williamsport, PA 17814, USA; smithr@lycoming.edu; 2 Department of Biology, University of Mississippi, 504 Shoemaker Hall, Oxford, MS 38677, USA

**Keywords:** riparian canopy, Plecoptera, Trichoptera, vertical migration, adult stream insects, streams

## Abstract

**Simple Summary:**

Stream insects are essential components of aquatic and terrestrial ecosystems, but fewer studies have examined the terrestrial stage compared to the aquatic stage. Most adult stream insects are flight capable, which allows for short- and long-distance movements away from the stream channel into riparian or upland habitats. This study examined if adult stream insects migrate vertically into riparian forest canopies above the stream. We found a meaningful number of adult Trichoptera and Plecoptera in the forest canopy above the stream that was comparable to horizontal migrations. This result demonstrated that adult stream insects utilize riparian forest canopies and that numerous avenues for additional basic and applied research on terrestrial–aquatic linkages exist. Discovering how riparian canopy habitats are important for stream insects and how stream insects are linked to canopy ecosystems can inform restoration and conservation actions.

**Abstract:**

Stream insects are essential components of aquatic and terrestrial ecosystem structure and function. Terrestrial stages are important components of terrestrial food webs, and flight-capable individuals are responsible for long-distance dispersal. Horizontal migrations by flying or crawling adults away from stream channels that link insects to riparian food webs and movements across catchment boundaries are well established through empirical research, but studies examining vertical migration of adult stream insects into forest canopies are generally lacking. This study focused on differences in adult Plecoptera and Trichoptera abundance at ground level versus the riparian canopy and differences in abundances among summer and autumn sampling periods to empirically demonstrate use of canopy ecosystems by stream insects. Malaise traps at ground level and canopy traps placed 8 to 10 m above the stream at four sites in the Mosquito Creek watershed (Pennsylvania) were used to examine vertical migration. Larval assemblages were collected and compared to adult assemblage to investigate patterns of local migration in the catchment. We found significantly more stream insects at ground level than in the forest canopy for Trichoptera, Plecoptera, and all individual plecopteran families, but a meaningful number of individuals were found in the riparian canopy. Canopy abundances were similar to abundances captured in adjacent ground-level habitats in other studies. Comparisons of adult and larval abundances among sites, taxa, and stages indicated site- and taxon-specific patterns for vertical movement into riparian canopies. Demonstrating that adult stream insects utilize riparian forest canopies indicates that riparian forest conservation should be prioritized over reforestation and that several potential research questions exist to inform riparian management.

## 1. Introduction

Stream insects play important roles in maintaining aquatic and riparian ecosystem structure and function [1,2] and are important components of aquatic and terrestrial food webs [3]. Many stream insects have complex life cycles that include aquatic and terrestrial stages. Adults for most taxa of Plecoptera and Trichoptera in temperate regions emerge during the spring and summer, but some taxa emerge in the autumn [4,5]. Insects commonly emerge as flight-capable adults, which allows them to disperse along stream channels and into upland areas. The abundance of stream insects is typically greatest close to or above the stream channel [6,7] due to increased success in finding suitable mates and oviposition sites [8]. Adults are preyed on by numerous vertebrate and invertebrate riparian and upland predators [9], and urban and agricultural land use in riparian and upland areas can cause poor survival and alter migration patterns of insects at ground level [10]. Empirical evidence also suggests that migration into upland areas at ground level by adults is limited [6,11], but adult insects can move across catchments through upland areas [12].

Yet, little evidence exists about how adult stream insects use forest canopies. Didham et al. [13] examined adult steam insect movement into forest canopies in upland areas and found that stream insects were more abundant in canopies than at ground level. However, the overall abundance of stream insects sampled was low due to sampling far from stream channels [13]. Adult stream insects can migrate away from streams into upland habitats but show the greatest abundance immediately next to the channel [6]. Thus, the abundance of adults in riparian forest canopies directly above the stream could be similar to abundances at ground level immediately adjacent to stream channels. Use of riparian canopies likely differs from upland habitats, and understanding patterns of canopy use near the stream channel where adults are most abundant can provide novel information about the natural history of stream insects, aquatic-terrestrial food web linkages [3], and management of riparian habitats [14].

Several factors can influence stream insect use of riparian canopy habitats including forest structure, environmental conditions, availability of mates, and predation risk [15]. High insect activity increases predator abundance in riparian zones [9,16], and riparian canopies may serve as refugia from certain riparian predators [17]. Adult stream insects may also use riparian canopies to complete their life cycle [18], which could include less inter or intraspecific competition for roosting sites. Moving into the riparian canopy could also provide access to wind currents for species with high wing-aspect ratio to passively disperse away from the stream [19,20].

Examining vertical movement of adult stream insects can also provide insight about the effects of human-dominated landscapes on adult assemblages. Artificial surfaces that reflect polarized light [21,22] and culverts that result in increased predation [23] are examples of anthropogenic structures that can hinder dispersal of adult stream insects [10]. Species evolved to disperse vertically into the canopy may avoid these obstacles, which could provide an advantage in urban and suburban landscapes. Yet, human-dominated landscapes commonly lack mature riparian forests along streams [24], and the lack of riparian vegetation may discourage vertical movements.

This study examined if adult stream insects utilized riparian canopies by comparing stream insect abundance and assemblage composition at ground level and in riparian canopies above stream channels among summer and autumn sampling periods. We hypothesized that stoneflies and caddisflies prefer ground-level habitats over riparian forest canopies, but a meaningful number of adults utilize canopy habitat. We also hypothesized that movement patterns and seasonal abundance patterns will be taxon specific. From these hypotheses, we predicted that (1) abundances for all taxa are greater at ground level than in the canopy, (2) relative abundances of individuals caught at ground versus canopy habitats will differ among taxa, (3) abundances for all taxa are greater in summer than autumn, and (4) taxa of larvae found in the stream reaches where adult sampling took place will match adult taxa found at the same site.

## 2. Materials and Methods

**Figure 1:**
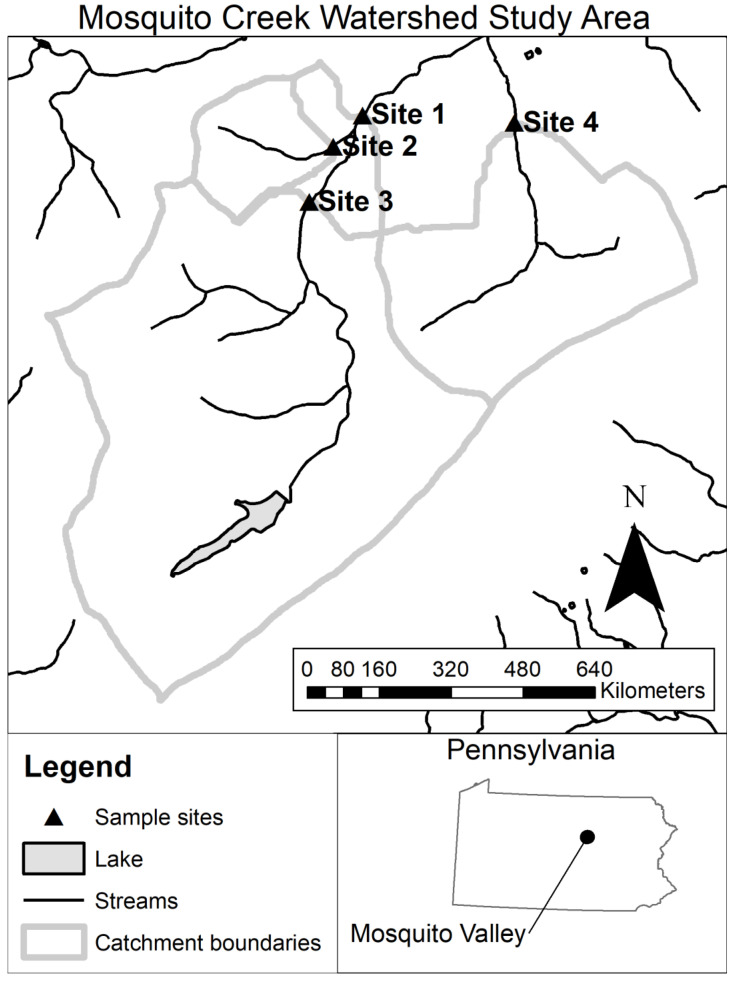
Map of study sites in the Mosquito Creek watershed.

All field work was conducted within the Mosquito Creek watershed in Pennsylvania located in the piedmont physiographic province (Figure 1). Samples were collected at four stream reaches from three different streams (Figure 1; Table 1). Stream reaches sampled were 1st, 2nd, and 3rd order but had similar characteristics. Sub-catchment size ranged from 1.6 to 19.1 km^2^. The overall catchment for Mosquito Creek is primarily forested (Table 1) but also contained agricultural land use upstream of Sites 1 and 3. The catchment’s forest was composed of a mix of conifers and hardwoods.

Stream width ranged from 3.9 to 5.7 m and depth ranged from 13.4 to 28.1 cm. Habitat was dominated by boulder (70–90%) or cobble substrates (60–80%; Table 1). Large woody debris and undercut banks were present at all sites and aquatic vegetation and leaf packs were present at two and one sites, respectively (Table 1).

**Figure 2:**
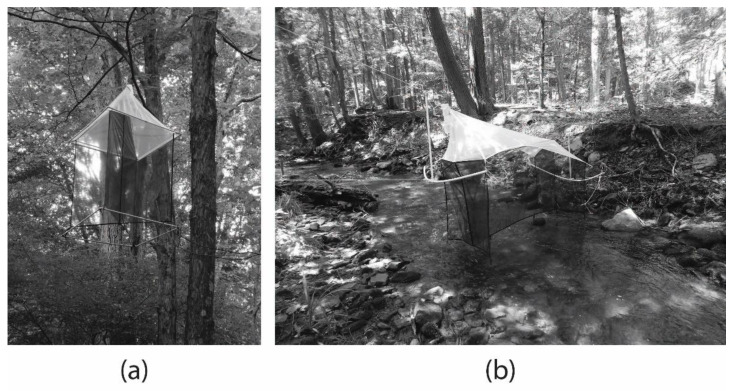
Images of a (**a**) malaise canopy trap and (**b**) Towns-style ground-level malaise trap. These are referred to as “canopy” and “ground” traps, respectively, for this study.

Townes-style malaise [25] and malaise canopy traps (Sante Traps, Lexington, KY, USA) were used to measure adult stream insect activity at ground level and in the forest canopy, respectively (Figure 2), and are referred to as “ground” and “canopy” traps for this study. The traps collected all terrestrial and aquatic flying insects, but this study focused on Plecoptera (stoneflies) and Trichoptera (caddisflies) with an additional focus on plecopteran families [26]. This study focused on stonefly families because they are associated with healthy stream ecosystems, are common indicators of stream health, were known to use riparian vegetation, and were ubiquitous throughout our study location (see [4] for more information on Plecoptera biology and life history). Previous research demonstrated that flight intercept traps (i.e., malaise traps) were efficient for trapping adult caddisflies and stoneflies, e.g., [20].

### 2.1. Aquatic Habitat Assessment

Aquatic habitat assessments were conducted in the summer of 2017 during a pilot study at the same reaches sampled for adults in 2018. Measurements of habitat and physiochemical properties for each site were conducted within a 40 m reach centered at the ground trap location. Habitat within the reaches was not expected to change for the duration of this study given the high percent of forested land cover in the catchment and flow control procedures used for managing drinking water supplies by the Williamsport Municipal Water Authority. Substrate type, embeddedness, flow type, thalweg depth (deepest part of the channel where current is the greatest), habitat, and wetted stream width were recorded at 11 transects spaced 4 m apart within the 40 m reach. Visual assessments were conducted to estimate the benthic substrate type(s) that comprised over 50% of the stream bottom. Substrates included silt, sand (<2 mm and granular), gravel (2–10 mm), pebble (1–6.4 cm), cobble (6.4–25.6 cm), boulder (>25 cm), and bedrock. Visual assessments were used to determine which flow type, riffle, run, or pool, comprised at least 50% of the habitat at each transect. A visual assessment determined the presence or absence of habitat types at each transect. Habitat types included logs, woody debris, aquatic vegetation, root balls, debris dams, muck, leaf packs, backwater areas, and undercut banks.

### 2.2. Sampling Protocol

Ground traps were placed directly above the stream channel at the middle of the 40 m reach to capture insects flying at or near the surface of the stream. Ground traps are open on two sides (collecting insects flying up and downstream) and at the bottom (collecting insects emerging from the stream; Figure 2). Traps were 1.9 m in width, 1.1 m in height on the front side with collecting jar, and 0.75 m in height on the back side. Frames were constructed of PVC pipe and attached to trees near the stream using ropes, and the traps were attached to frames using bungee cords. Ground traps were adjusted using the ropes to sit approximately 2.5 cm above the stream surface, but the actual distance above the stream changed as stream levels fluctuated. Traps were checked when samples were collected and adjusted to prevent any part of the trap from becoming submerged, but the bottom of traps touched the water or were submerged a few centimeters during high flow events. Individual adult insects were collected in 500 mL sampling jars attached to the top corner of the traps filled with approximately 250 mL of 70% ethanol. Bottles were unscrewed, the specimens removed, and the sample bottles replaced after being refilled with 250 mL ethanol during each collection.

Canopy traps were open on all four sides and on the bottom and maintained their shape due to PVC plastic frames. Each single opening was 0.9 m in width and 1.2 m in height. Canopy traps were placed up or downstream of the ground trap at the closest suitable location to hang directly over the stream channel from overhanging trees at the desired height of approximately 8 to 10 m (canopy trap heights for Sites, 1, 2, 3, and 4 were 8.0, 8.7, 8.2, and 9.6 m, respectively). Canopy traps collected insects from all directions laterally and insects moving up from below (Figure 2). The sample bottle was at the apex of the trap and filled with approximately 250 mL of 70% ethanol. Each trap was suspended in the canopy by 45 kg test braided fishing line attached by a 68 kg snap-swivel. The other end of the fishing line was attached to a tree at ground level to allow for the trap to be lowered for sample retrieval. For each collection date, sample bottles were removed, the contents emptied into another 500 mL jar, and the trap’s sample jar refilled with ethanol and replaced. The trailing end of the fishing line had a second swivel at a fixed location on the line to secure it at ground level so the trap was raised to the same height after each collection.

Ground and canopy traps were left out for 14 consecutive days during summer (18 June to 2 July) and autumn (30 August to 13 September) sampling periods. Traps were checked for damage and repaired, and all spiders were removed during each collection. Collections were made and traps were reset every three or four days during the summer sample period (emptied four times) and every five or six days during the autumn sample period (emptied three times). A total of four ground and four canopy samples were collected at each site for a total of 32 samples during summer. A total of three ground and three canopy samples were collected at each site for a total of 24 samples during autumn, but the final canopy trap sample from Sites 1 and 3 were unusable (i.e., only 22 usable samples from the autumn period). The tree the canopy trap was attached to fell over at Site 1, and the canopy trap at Site 3 was tangled in a branch, which caused the collecting jar to be filled with water at the time of collection. The contents of adult samples were sorted under magnification to remove all Plecoptera and Trichoptera. Plecoptera were identified to family using a comprehensive key to aquatic insects of North America [27].

A D-net with a 30.5 cm opening and 500 µm mesh net was used to collect larval aquatic insects from the stream to characterize the in-stream assemblage on 2 July 2018. A 10-kick composite sample was taken from 10 randomly stratified locations from each habitat type in the proportion that the habitat type existed within the 40 m stream reach. The benthos was disturbed for 10 s per individual subsample (i.e., per individual kick). A multiple habitat sampling method was employed to ensure the largest diversity was found [28]. One to three subsamples were collected together and then transferred to a 355 µm sieve where large debris (e.g., leaves and sticks) were rinsed and removed from the sample. The sample was then transferred to a 500 mL sampling bottle, preserved with 70% ethanol in the field, and returned to the lab for sorting. All larval Plecoptera and Trichoptera were removed and enumerated. All Plecoptera were identified to family using a comprehensive key to aquatic insects of North America [27] and enumerated.

### 2.3. Analysis

The relative difference in the abundance of adult Trichoptera and Plecoptera and plecopteran families between ground or canopy habitats was calculated to determine the use of canopy habitats and seasonal differences in abundance. Statistical comparisons of abundance were performed using a Wilcoxon sign rank test used for paired data that are not normally distributed (i.e., count data). All adult subsamples taken over the 14 day period for each site and season were combined for analysis (i.e., an observational unit was the total abundance caught over a 14 day period for a single site in a single season). A total of eight pairs (16 total observational units) were used for all analyses. During the autumn sampling period, however, one subsample from the canopy trap was lost at Sites 1 and 3. The total abundance collected over 9 days of sampling was standardized to 14 days for analysis. Analysis examining ground versus canopy paired samples based on season and site (i.e., the canopy trap sample at Site 4 in the summer was paired with the ground trap sample at Site 4 in the summer). A paired test was used to keep daily weather (that may discourage activity) and the overall reduced abundance in the autumn from biasing our results. Chloroperlidae, Peltoperlidae, and Perlidae had 0 abundance in autumn ground and canopy traps, and no statistical significance is reported for comparisons of ground versus canopy abundance to avoid presenting biased results. Comparisons of abundance among seasons paired samples based on trap type and site (i.e., the summer canopy trap at Site 4 was paired with the autumn canopy trap at Site 4). A lack of significant difference in abundance among trap types or among seasons would indicate no preference for ground versus canopy habitat or no seasonal difference respectively. All statistical tests were completed using the R-Statistical Program (v 4.0.4; The R Foundation for Statistical Computing) [29].

An examination of potential density dependent patterns of vertical migration of adults into the canopy was performed by calculating Spearman rank-order correlation coefficients for ground and canopy trap abundances paired by site and season for each plecopteran family. Data standardized to 14 day abundances (described above) were used in this analysis. Correlations among paired canopy and ground abundances would indicate that canopy abundances fluctuate with ground-level abundances possibly due to density dependent factors driving patterns of vertical migration. The correlation analysis was performed using the R statistical program (v 4.0.4; The R Foundation for Statistical Computing) [29].

Comparisons of adult and larval assemblage composition were performed by comparing relative abundance among sites, seasons, and trap types. Comparisons of relative abundance among ground and canopy assemblages can demonstrate taxon-specific and site-specific differences in vertical migration and where adults move locally following emergence. Collections of adults from ground and canopy traps and of larvae from the benthos likely have different capture efficiencies due to the environment and sampling method. Thus, a formal analysis of composition using multivariate methods was unnecessary given the scope of this study.

## 3. Results

Overall, an estimated 14,821.78 adult stream insects were included in the analysis (abundances were rescaled to 14 day periods due to sample loss as described in the methods). Trichoptera were more abundant overall (*n* = 11,331.12) than Plecoptera (*n* = 3490.66; Table 2). Trichoptera were more abundant than Plecoptera in ground traps (*n* = 9191 and *n* = 2934, respectively) and canopy traps (*n* = 2140.12 and *n* = 556.66, respectively). Trichoptera were more abundant than Plecoptera in all samples except for the summer ground trap at Site 2 and autumn ground trap at Site 3. Plecoptera were at least twice as abundant in the ground trap at Site 2 than the other three sites in the summer, but the ground trap at Site 3 had the greatest number of Plecoptera among all sites in autumn.

The total abundance of adults aggregated across the full sample period (14 days) during the summer sampling period in ground traps ranged from 300 to 1092 and 583 to 4087 for Plecoptera and Trichoptera, respectively (Table 2). The total abundance of adults aggregated across the full sample period (14 days) during the summer sampling period in canopy traps ranged from 41 to 156 and 146 to 733 for Plecoptera and Trichoptera, respectively (Table 2). The total abundance of adults aggregated across the full sample period (14 days) during the autumn sampling period in ground traps ranged from 84 to 238 and 101 to 299 for Plecoptera and Trichoptera, respectively (Table 2). Total abundance aggregated across the full sample period (14 days) of adults during autumn sampling period in canopy traps ranged from 13 to 48.22 and 41 to 92 for Plecoptera and Trichoptera, respectively (Table 2).

For plecopteran families, Perlodidae was the most abundant (*n* = 1251.12) and Peltoperlidae was the least abundant (*n* = 27) for all samples combined (Table 3). Perlodidae was the most abundant in all ground traps combined (*n* = 1148), and Leuctridae was the most abundant in all canopy traps combined (*n* = 208.11). Perlodidae was also the most abundant in all summer ground traps combined (*n* = 1031), and Nemouridae was the most abundant in all summer canopy traps combined (*n* = 111). Leuctridae was the most abundant in autumn ground (*n* = 374) and autumn canopy (*n* = 116.11) traps combined. The highest abundances in a single ground and canopy trap sample were 487 (Perlodidae) and 49 (Nemouridae) individuals, respectively. Zero abundance values were more common for plecopteran families in the autumn (27) than summer (2) and were more common in canopy (16) than ground (13) traps (Table 3).

Perlodidae was the most abundant family in all summer ground traps except at Site 3. Leuctridae was the most abundant family in all autumn ground and canopy traps. Perlodidae were generally less abundant in canopy than ground traps. The greatest abundance of Chloroperlidae was found during the summer sampling period in the ground trap at Site 2. Zero individuals were captured for Chloroperlidae, Peltoperlidae, and Perlidae during the autumn sampling period for both trap types. Nemouridae had zero individuals captured in the ground trap at Site 4 and in canopy traps at Sites 2 and 4 during the autumn sampling period. Peltoperlidae had zero individuals captured in canopy traps at Sites 1 and 2 during the summer sampling period.

### 3.1. Analysis of Ground Level versus Canopy Preference

**Figure 3:**
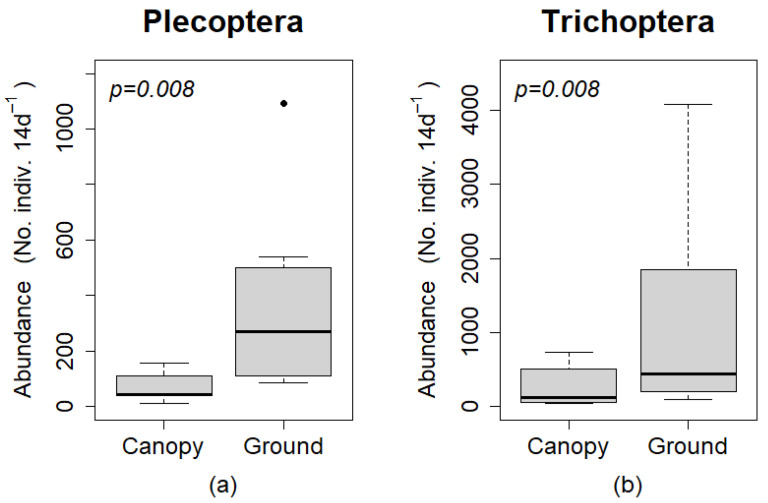
Box plots comparing estimated abundances (No. individuals per 14 day period) of (**a**) Plecoptera and (**b**) Trichoptera among canopy and ground traps. Boxes represent upper and lower quartiles, and whiskers extend to values no greater than or less than 1.5 times the inter-quartile range of the upper and lower quartiles, respectively. Points (•) are outliers outside the values of the whiskers. Statistical comparisons were performed using a Wilcoxon sign rank test.

The abundance of Plecoptera and Trichoptera were significantly higher in ground than canopy traps (*p* = 0.008 for both orders; Figure 3). The abundance of individuals per 14 day period was greater in ground than canopy traps for every paired sample (same season and site) for both orders.

**Figure 4:**
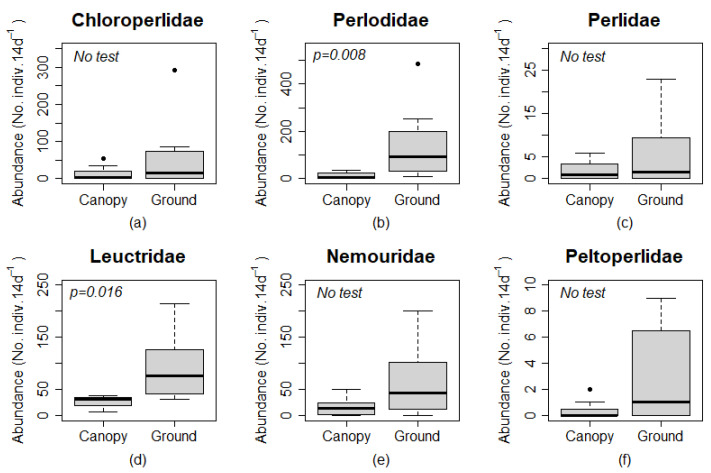
Box plots comparing abundances (No. individuals per 14 day period) of plecopteran families (**a**–**f**) among canopy and ground traps. Box plots include zero data, and statistical significance are only shown for taxa without paired zero abundances among trap types. Boxes represent upper and lower quartiles, and whiskers extend to values no greater than or less than 1.5 times the inter-quartile range of the upper and lower quartiles, respectively. Points (•) are outliers outside the values of the whiskers. Statistical comparisons were performed using a Wilcoxon sign rank test.

Measures of stonefly abundance was greater in ground than canopy traps for all pairs of samples from each site and season combination except for Leuctridae (*n* = 31 and *n* = 30 for canopy and ground traps, respectively) and Perlidae (*n* = 4 and *n* = 3 for canopy and ground traps, respectively) in summer at Site 4. Site 4 also included all three instances where the abundance in canopy traps was at least 50% of the abundance in ground level traps for Chloroperlidae, Nemouridae, and Peltoperlidae. Chloroperlidae and Peltoperlidae were not found at ground level or in the canopy for any autumn sample, and Perlidae was only found at ground level for all autumn samples. Family-level analysis of Plecoptera found a significantly greater number of adults in ground than canopy traps for Perlodidae (*p* = 0.008) and Leuctridae (*p* = 0.016; Figure 4). The abundance of Chloroperlidae, Perlidae, and Peltoperlidae were 0 in all 4 canopy and ground traps in the autumn, and Nemouridae were 0 in canopy and ground traps at Site 4 in the autumn. Test of statistical significance were not completed on these taxa, but the numerical difference among samples indicated that more individuals were found at ground level than in the canopy for these families.

**Figure 5:**
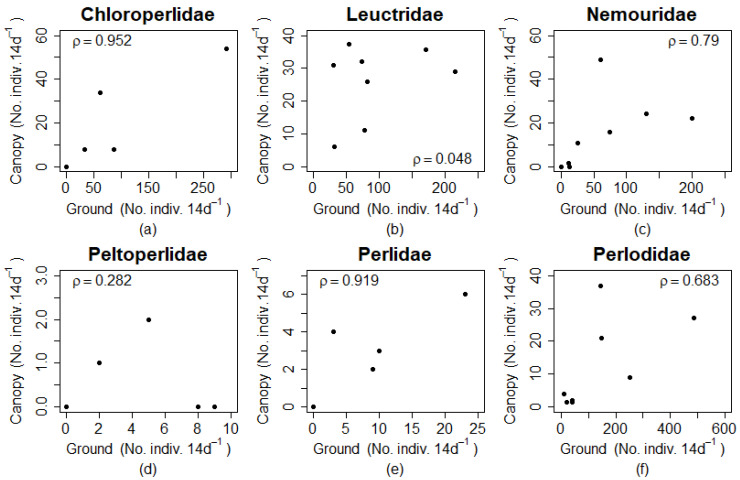
Scatter plot of paired ground and canopy trap abundances for each site and season individually for all (**a–f**) plecopteran families. Spearman rank-order correlation coefficients (ρ) indicate the strength of correlation for paired samples and are reported for each family.

The correlation analysis showed strong correlations among ground and canopy abundances for Chloroperlidae, Nemouridae, and Perlidae (Figure 5). Leuctridae and Peltoperlidae were not strongly correlated. Perlidae and Peltoperlidae had low abundances but different ρ values indicating that correlations where not dependent on overall abundance.

### 3.2. Analysis of Seasonal Differences in Abundance

**Figure 6:**
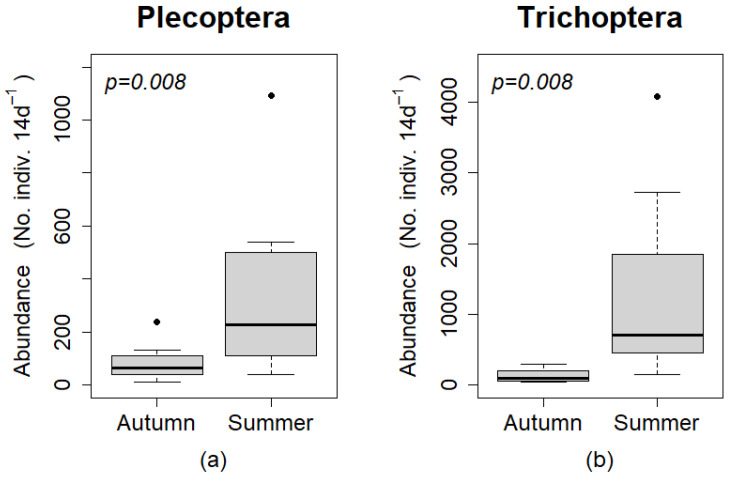
Box plots comparing abundances (No. individuals per 14 day period) of (**a**) Plecoptera, and (**b**) Trichoptera among sampling period. Boxes represent upper and lower quartiles, and whiskers extend to values no greater than or less than 1.5 times the inter-quartile range of the upper and lower quartiles, respectively. Points (•) are outliers outside the values of the whiskers. Statistical comparisons were performed using a Wilcoxon sign rank test.

A significantly greater number of individuals for Plecoptera and Trichoptera, were captured during the summer sampling period than the autumn sampling period (*p* = 0.008 for both orders; Figure 6). The abundance of individuals per 14 day period was greater in the summer sampling period than the autumn sampling period for every paired sample (same trap type and site) for both orders.

**Figure 7:**
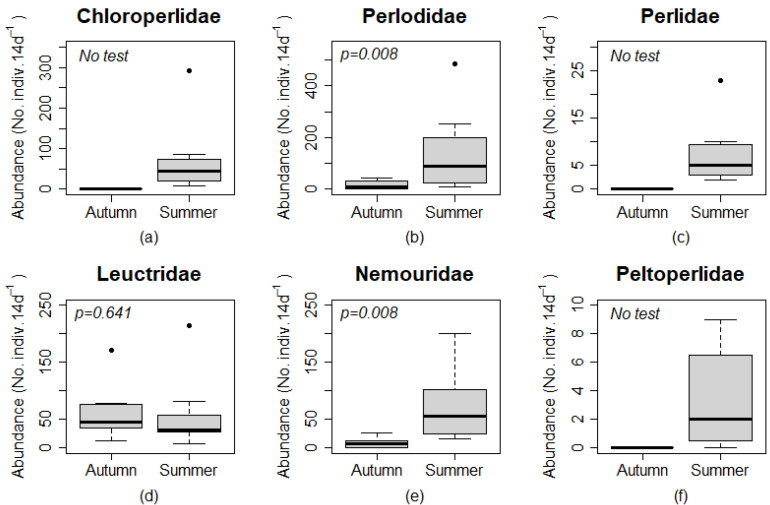
Box plots comparing abundances (No. individuals per 14 day period) of plecopteran families (**a–f**) among sampling period. Boxes represent upper and lower quartiles, and whiskers extend to values no greater than or less than 1.5 times the inter-quartile range of the upper and lower quartiles, respectively. Points (•) are outliers outside the values of the whiskers. Statistical comparisons were performed using a Wilcoxon sign rank test.

No individuals were caught during the autumn sampling period for Chloroperlidae, Peltoperlidae, and Perlidae (Table 3), which demonstrated an obvious difference in abundance among seasons without the use of a statistical test. Perlodidae (*p* = 0.008) and Nemouridae (*p* = 0.008) had a significantly greater number of individuals caught during the summer sampling period than the autumn sampling period (Figure 7). Leuctridae abundance among seasons was not significantly different (*p* = 0.641; Figure 7).

### 3.3. Adult, Larval, and Benthic Assemblage Composition

**Figure 8:**
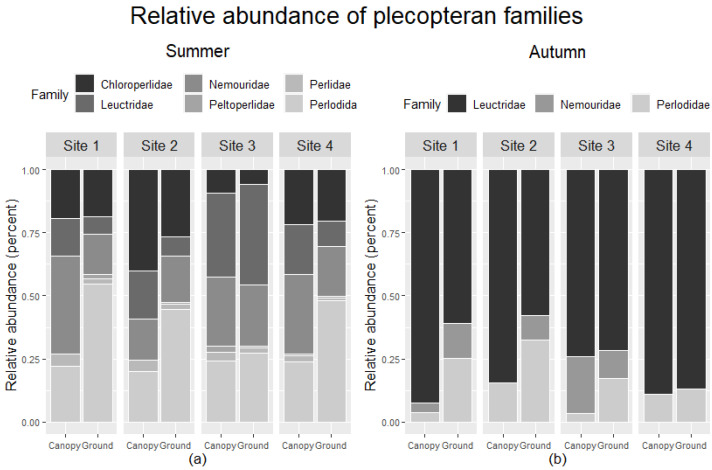
The relative abundance of plecopteran families found in canopy and ground traps among sites for the (**a**) summer and (**b**) autumn collections.

Comparing relative abundance in ground and canopy trap samples across sites indicated taxon and site-specific differences in vertical migration (Figure 8). During summer, Site 3 had similar relative abundances for plecopteran families, and the other sites generally had higher relative abundances of Perlodidae in ground traps than in canopy traps. Nemouridae had a larger relative abundance in the canopy than ground traps at Sites 1 and 4. Site 3 had a comparably large relative abundance of Leuctridae in the canopy and ground traps than other sites. Chloroperlidae had a similar relative abundance among canopy and ground traps across all sites but had the highest relative abundance at Site 2. Peltoperlidae and Perlidae had similar relative abundances among trap types but also had overall low abundance. Autumn samples only included three families. Perlodidae had higher relative abundance in ground than canopy traps at Sites 1, 2, and 3. The relative abundance of Leuctridae was higher in canopy than ground at Sites 1 and 2. Nemouridae relative abundance was higher in ground than canopy traps at Sites 1 and 2, lower in ground than canopy traps at Site 3, and absent from Site 4.

**Figure 9:**
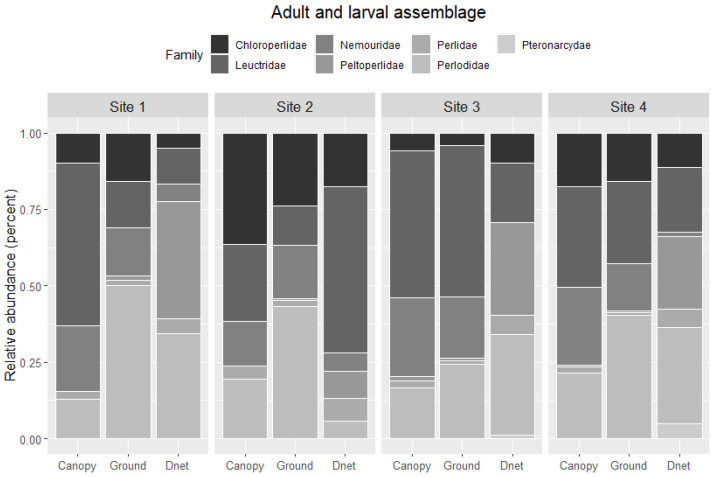
Stacked bar chart representing relative abundance and assemblage composition at each site for adult plecopteran taxa in ground and canopy traps and larval plecopteran taxa in benthic samples. Abundances for ground and canopy traps from summer and autumn samples are combined for this analysis.

The relative abundance of larvae in benthic samples generally did not match the relative abundance of adults in ground or canopy traps (Figure 9). Larval Peltoperlidae had high relative abundance at Sites 1, 2, and 4 but low adult relative abundance in ground and canopy traps. Similarly, adult Nemouridae relative abundance was generally greater for ground and canopy traps than in benthic samples. Leuctridae also had a higher relative abundance in the benthos than in the ground or canopy trap samples at Site 2, and Leuctridae relative abundance for the benthos sample at Site 3 was lower than the ground and canopy samples. Perlodidae had a larger relative abundance in the ground trap than the canopy trap or benthic sample at Sites 1 and 2, but Perlodidae had consistent relative abundance across sites. Chloroperlidae had higher relative abundance for all sample types at Site 2 than the other three sites. Pteronarcyidae was not caught as adults in ground or canopy traps but was found in the benthos at Sites 2, 3, and 4.

## 4. Discussion

Research on adult stream insect activity has focused on analyzing abundance at ground level with minimal research conducted on vertical migration into riparian forest canopies. This study examined how abundances of adult Trichoptera and Plecoptera and plecopteran families differed among canopy and ground-level traps. Additionally, this study examined seasonal differences in abundance and how composition differed among canopy, ground-level, and benthic habitats. While adult stream insects generally preferred staying at ground level, biologically meaningful abundances of adults were documented in forest canopies indicating vertical migration. Examination of spatial patterns in plecopteran family assemblages indicated that species and site-specific factors may determine vertical patterns of abundance. As expected, adults were more abundant in the summer than autumn.

### 4.1. Ground Level versus Canopy Abundance

Overall, caddisflies, stoneflies, and individual plecopteran families preferred to stay near ground level above the stream, but the overall results also conclusively indicated that a meaningful portion of the adult assemblage is using forest canopy habitats near streams. Didham et al. [13] previously found a larger abundance of Trichoptera in forest canopies than at ground level and more Plecoptera at ground level than in the canopy. This study found a significantly greater number of Plecoptera and Trichoptera at ground level than in the canopy. Didham et al. [13], however, sampled in upland areas and found a substantially lower overall abundance than this study (e.g., 204 Trichoptera and Plecoptera individuals in the canopy caught over 70 days using 317 60 cm by 23 cm sticky traps versus 2696.8 standardized individuals in the canopy from two orders over 28 days of sampling).

The abundances of Plecoptera and Trichoptera found in the canopy of this study are comparable to abundances found in adjacent riparian forests at ground level [30]. Petersen et al. [30] found 72% fewer stonefly adults and 53% fewer caddisfly adults in ground traps placed 0 m away from the stream in forested catchments and 93% fewer stoneflies in all samples taken 5 to 75 m from the stream (caddisfly abundance increased due to one taxa). We found 82% and 75% fewer stoneflies and 77% and 69% fewer caddisflies in the canopy than at ground level for the summer and autumn, respectively. The similar decrease in abundance suggests the ecological importance of adult stream insects in canopy habitats could be similar to ground-level riparian ecosystems [3].

Similar to ground-level riparian habitats, canopy habitats can provide substrates for finding mates, roosting locations, refugia from predators, etc., and species-specific adaptations for vertical migration may occur. Certain plecopteran taxa feed as adults on algae, lichens, nectar, pollen, and other resources that may occur in the canopy [14,31]. For example, forests surrounding the study sites included *Liriodendron tulipifera* (Tulip Poplar), which have large flowers in the canopy that could serve as a food source for adults. Microclimate conditions in canopy habitats may also be ideal for certain species [14,32]. Sunlight in canopy habitats differs from sunlight in dense understory habitats. Sunny areas may attract certain species, but low-humidity and high-temperature microclimates can increase the mortality of adults for certain species [32].

The observed difference in abundance among trap types may be the result of discrepancies in trap capture efficiencies due to trap design and the biology of the organisms. Our use of flight intercept traps provided no information to assess how adults moved into the canopy through any process other than flight, but stoneflies emerge as larvae that can crawl into canopy habitats prior to transitioning into a flight-capable adult [4]. Additionally, ground traps are likely more efficient at capturing adult stream insects than canopy traps due to their placement and size. Ground traps are placed directly along the stream corridor where most stream insects travel and are designed to catch insects moving up and down the stream channel [7]. Even though canopy traps were placed directly over the stream channel, adults in the canopy are likely more dispersed than individuals aggregated above the stream at ground level. A lack of efficiency of canopy traps would provide a conservative estimate of adult stream insect canopy use, and further study using similar trap types may demonstrate greater canopy use than we demonstrated in this study.

Even though canopy traps were not placed above ground traps, ground traps may have inflated abundance due to catching emerging individuals that may otherwise be moving vertically towards the canopy in addition to individuals flying vertically along the stream channel. Testing the relative contribution of flying adults that intercept ground traps and emerging adults from the stream to abundance in ground traps is an important research question given the common use of ground traps to measure adult stream insect assemblages.

### 4.2. Seasonal Difference in Abundance

Many stream insect larvae use autumn, winter, and spring to grow and store enough resources to complete metamorphosis prior to emergence [8]. Seasonal abundance data for adult taxa examined in this study conformed to expectations that summer abundances are greater than autumn abundances. The lack of a significant difference among seasons for Leuctridae, however, is likely due to multiple species emerging at different seasons. Detecting species-specific phenological differences requires species-level identifications, which was beyond the scope of this study. Further study to document the timing of life history processes is needed to inform conservation of stream insect biodiversity, e.g., [33].

### 4.3. Taxon- and Site-Specific Differences among Plecopteran Families

Measures of absolute and relative ground and canopy trap abundance showed taxon-specific differences in the use of the canopy among plecopteran families. Perlodidae was the most abundant in all ground traps combined (*n* = 1148), but Leuctridae was the most abundant in all canopy traps combined (*n* = 208.1). Perlodidae had small relative canopy trap relative abundance compared to ground level, and Leuctridae had high relative canopy trap relative abundance compared to ground level. These results suggested that individuals from the family Perlodidae overall had less of a preference for migrating into the canopy than other taxa, and individuals from the family Leuctridae had a greater affinity for vertical migration than other families. The mechanism for this difference, however, is not clear from our results and is an area for future study including species identifications to provide additional information about mechanisms for vertical migrations.

The relative abundance of individuals in the canopy could result from density dependence where a high abundance of individuals at ground level triggers vertical migrations. The results of the correlation analysis suggested that Chloroperlidae, Perlidae, and Nemouridae vertical migrations may be density dependent, but Leuctridae and Peltoperlidae are not. The high ρ value for Chloroperlidae and Perlidae, however, is due partly to the lack of adults found in either ground or canopy traps in the autumn (i.e., paired zero values for half the samples). Density dependent vertical migration could have evolved to assist with finding roosting sites but could also be maladaptive if it decreases chances of finding mates who are aggregated directly above the stream. Density dependent vertical migration could also be a response to crowding to move away from the stream. Again, these are all potential research questions for future study.

Site 4 consistently had the largest relative abundance of adults in the canopy. Site-specific factors such as riparian tree species and age of the forest may directly alter canopy use or indirectly alter canopy use by changing riparian predators [34]. Ground-level riparian vegetation sometimes covered the stream channel at Site 4 but not the reaches at the other sample sites. A dense understory growing over the stream channel could result in numerous differences in local habitat such as reduced polarized reflected light observed by adults that failed to trigger aggregation above the channel at ground level [21,22].

The overall lack of congruence among larval and adult assemblages could be the result of local immigration and emigration by adults. Adults moving away from or to a site may be in search of oviposition sites, mates, or other structures needed for completing life history processes [8]. Site 2 had a greater relative abundance of Leuctridae larvae than adults, which could indicate that Site 2 had different in-stream habitat than the other sites. Site 2 had the smallest catchment and was the shallowest stream. Additionally, Site 2 had more fine sediment than other sites even though it was dominated by cobble substrates. Adults of certain taxa emerging from surrounding reaches may avoid Site 2, or adults may emerge from Site 2 and immediately leave in search of better habitat such as faster moving water or reaches with low sediment in the benthos. Both processes would have resulted in the observed differences between larval and adult relative abundance.

### 4.4. Applications

Riparian areas are commonly deforested in landscapes with urban and agricultural land use [35]. Riparian deforestation increases stream bank erosion, decreases benthic habitat quality, decreases riparian habitat quality, and decreases riparian and stream biodiversity [36]. Stream insects are essential for terrestrial and aquatic food webs [3], and benefits to insect assemblages from riparian forest conservation will have secondary benefits for aquatic and terrestrial species. The results of this study suggested that future work to examine characteristics of canopy habitats could indicate novel approaches for effective riparian forest conservation.

Planting trees along stream banks can benefit a stream by reducing nutrient inputs, liming bank erosion, and improving aquatic habitat [36], and is a common approach to stream restoration. Riparian reforestation may also benefit adult insect survival for species that utilize the riparian canopy. Restoration projects that plant trees, however, may not provide benefits to adult stream insects until riparian forests grow mature canopies [15]. Additionally, the understory density of immature forests may also alter patterns of vertical migrations. While replanting trees has overall benefits to streams and direct benefits for stream insect adults, the extended time until a benefit is realized for adult insects demonstrates the importance of preserving mature riparian forests.

## 5. Conclusions

The abundance of adult stream insects is highest at ground level above the stream, but the use of surrounding terrestrial habitats is important for population persistence and for supporting terrestrial food webs. While limited in scope, this study demonstrated that adult stream insects migrate vertically into riparian forest canopies and provided a foundation for future studies to examine basic and applied questions examining the ecology of riparian canopy ecosystems. Research is needed to examine evolutionary drivers of vertical migration into the canopy, predator–prey interactions, and the natural history of stream insects. Mechanisms determining vertical migrations can also provide insight about conserving and restoring riparian forests and stream ecosystems.

## Figures and Tables

**Table 1:** Catchment characteristics and stream habitat conditions at the four sample sites. Depth and width values are averages. (V = aquatic vegetation; B = undercut bank; LW = loose woody debris; LP = leaf pack.)

Site	Lat/Lon	Catchment Size (km^2^)	Catchment % Forest	Depth (cm)	Width (m)	Dominant Benthic Substrate	Dominant Habitat
1	41.191959/-77.067351	19.1	89%	27.6	5.2	Boulder (70%)	V, B, LW
2	41.188871/-77.071236	1.6	100%	13.4	3.9	Cobble (80%)	B, LW, LP
3	41.183346/-77.074472	16.4	88%	28.1	5.7	Boulder (90%)	B, LW
4	41.191121/-77.047209	6.3	100%	20.5	4.1	Cobble (60%)	V, B, LW

**Table 2:** Abundances of Plecoptera and Trichoptera between trap type and sampling periods per 14 days. Autumn canopy traps at Sites 1 and 3 sampled for 9 days due to trap failures, and abundances were rescaled to abundance per 14 days.

Period	Trap	Site	Plecoptera	Trichoptera
Summer	Canopy	1	41	332
Summer	Canopy	2	135	146
Summer	Canopy	3	87	733
Summer	Canopy	4	156	681
Summer	Ground	1	460	2730
Summer	Ground	2	1092	583
Summer	Ground	3	540	978
Summer	Ground	4	300	4087
Autumn	Canopy	1	40.44	43.56
Autumn	Canopy	2	13	41
Autumn	Canopy	3	48.22	71.56
Autumn	Canopy	4	36	92
Autumn	Ground	1	87	299
Autumn	Ground	2	133	236
Autumn	Ground	3	238	101
Autumn	Ground	4	84	177

**Table 3:** Abundances of the six plecopteran families among trap types and sampling periods.

Site	Chloroperlidae	Leuctridae	Nemouridae	Peltoperlidae	Perlidae	Perlodidae
Summer canopy traps						
1	8	6	16	0	2	9
2	54	26	22	0	6	27
3	8	29	24	2	3	21
4	34	31	49	1	4	37
Summer ground traps						
1	86	31	74	8	9	252
2	292	81	200	9	23	487
3	32	215	130	5	10	148
4	61	30	60	2	3	144
Autumn canopy traps						
1	0	37.33	1.56	0	0	1.56
2	0	11	0	0	0	2
3	0	35.78	10.89	0	0	1.56
4	0	32	0	0	0	4
Autumn ground traps						
1	0	53	12	0	0	22
2	0	77	13	0	0	43
3	0	171	26	0	0	41
4	0	73	0	0	0	11

## Data Availability

Data for this study are available from the authors upon request and will be provided to the Williamsport Municipal Water Authority at the conclusion of this study.
